# Identification of genes for normalization of real-time RT-PCR data in breast carcinomas

**DOI:** 10.1186/1471-2407-8-20

**Published:** 2008-01-22

**Authors:** Maria B Lyng, Anne-Vibeke Lænkholm, Niels Pallisgaard, Henrik J Ditzel

**Affiliations:** 1 Department of Pathology, Odense University Hospital, Odense, Denmark; 2 Medical Biotechnology Center, University of Southern Denmark, Odense, Denmark; 3 Department of Oncology, Odense University Hospital, Odense, Denmark

## Abstract

**Background:**

Quantitative real-time RT-PCR (RT-qPCR) has become a valuable molecular technique in basic and translational biomedical research, and is emerging as an equally valuable clinical tool. Correlation of inter-sample values requires data normalization, which can be accomplished by various means, the most common of which is normalization to internal, stably expressed, reference genes. Recently, such traditionally utilized reference genes as GAPDH and B2M have been found to be regulated in various circumstances in different tissues, emphasizing the need to identify genes independent of factors influencing the tissue, and that are stably expressed within the experimental milieu. In this study, we identified genes for normalization of RT-qPCR data for invasive breast cancer (IBC), with special emphasis on estrogen receptor positive (ER+) IBC, but also examined their applicability to ER- IBC, normal breast tissue and breast cancer cell lines.

**Methods:**

The reference genes investigated by qRT-PCR were RPLP0, TBP, PUM1, ACTB, GUS-B, ABL1, GAPDH and B2M. Biopsies of 18 surgically-excised tissue specimens (11 ER+ IBCs, 4 ER- IBCs, 3 normal breast tissues) and 3 ER+ cell lines were examined and the data analyzed by descriptive statistics, geNorm and NormFinder. In addition, the expression of selected reference genes in laser capture microdissected ER+ IBC cells were compared with that of whole-tissue.

**Results:**

A group of 3 genes, TBP, RPLP0 and PUM1, were identified for both the combined group of human tissue samples (ER+ and ER- IBC and normal breast tissue) and for the invasive cancer samples (ER+ and ER- IBC) by GeNorm, where NormFinder consistently identified PUM1 at the single best gene for all sample combinations.

**Conclusion:**

The reference genes of choice when performing RT-qPCR on normal and malignant breast specimens should be either the collected group of 3 genes (TBP, RPLP0 and PUM1) employed as an average, or PUM1 as a single gene.

## Background

Prognostic and predictive molecular markers associated with breast cancer are allowing individualization of treatment, and quantitative real-time RT-PCR is frequently used to measure the expression of these markers. The advantages of this technique are numerous, including its ability to sensitively quantify specific mRNA despite small samples sizes or low numbers of mRNA [1-4]. Working with absolute quantities based on standard curves is time-consuming and laborious. A target gene can be analyzed much more easily and precisely by correlation to a stable independent parameter, i.e. directly proportional to the amount of mRNA and not influenced by factors such as hormones, cell cycle status, etc. This technique is termed 'normalization', and the prevailing method is the use of reference genes [5].

The primary advantage of using genes expressed within the cells investigated as reference genes is that they also function as endogenous controls, since they are exposed to the same conditions *in vivo *and *in vitro*, thereby providing a direct indication of the quantity and quality of the samples. Widely-used genes include β-actin (ACTB), glydecaldehyde-3-phosphate-dehydrogenase (GAPDH) and β-2-microglobulin (B2M). However, it has recently become clear that the expression of some of these genes may be modulated during cellular processes such as differentiation and cancer progression, and are also susceptible to hormonal influences [1,5] as exemplified by β2-microglobulin and GAPDH. B2M have been found to be influenced by factors present in brain cortices of human chronic alcoholics [6]. GAPDH have been reported to be regulated by oestradiol, showing a dose-dependent, statistically-significant, increase in expression in MCF-7 cells [7]. Despite this shortcoming, however, GAPDH continues to be utilized as a normalizer in investigations of breast cancer and cell lines by RT-qPCR.

It is therefore necessary to identify appropriate reference genes for each experimental set-up. This is especially important for studies investigating subtle differences in the analyzed sub-groups, since such studies are more sensitive to minor fluctuations of the reference gene(s) that could lead to an incorrect rating of the target gene expression levels. It is also now widely accepted that more than one reference gene is needed to reduce the sensitivity to degraded material and to varying amounts of input RNA [8,9].

Approximately 80% of all invasive breast cancers (IBCs) are estrogen receptor positive (ER+), meaning they express the ER above a cut-off value, usually 5–10%, as determined by immunohistochemistry (IHC). These patients are eligible for endocrine treatment such as Tamoxifen that specifically target the ER, or the newer aromatase inhibitors (AI) that target the aromatase enzyme,, preventing estrogen synthesis. Unfortunately, about 30% of these patients do not benefit from Tamoxifen treatment and reports of relapse after AI are emerging [10,11]. These results have prompted many studies to elucidate this "anti-estrogen resistance", and RT-qPCR is a widely used technique in such studies. Although the cancers in these patients express the ER at the protein level, which is one of the routinely applied predictive markers to date for this breast cancer sub-type, it is likely that there are underlying molecular defects that render them unresponsive either through *de novo *or acquired resistance. The desire to further sub-group these patients has led to investigation of gene expression, which has highlighted the need for stable, hormone-independent, normalizers.

Two computer programs that allow relative identification of reference genes have been developed for Microsoft Excel^® ^either as a Visual Basic Application tool (geNorm [12]) or as an Add-in (NormFinder [13]), which can be used to objectively pinpoint the appropriate genes. Briefly, geNorm ranks the genes according to the average pair-wise variation of a particular gene with all other genes, and also provides a measure of the minimum optimal number of reference genes to avoid the expense and 'noise' in the assay from using too many reference genes. NormFinder calculates the stability value for all candidate normalization genes, providing a rank order and direct estimation of expression variation. Moreover, it provides the option of defining sub-groups among the samples the reference genes are tested on, such as tumor grade, size, etc.

In this study, we systematically evaluated a panel of endogenously expressed genes to identify those that would be most useful in normalization of RT-qPCR data from ER+ IBCs. In addition, we investigated whether these genes might also be useful in ER- IBCs, normal tissue and breast cancer cell lines.

We identified a 3-gene group (TATA-box binding protein (TBP), Ribosomal protein, large, P0 (RPLP0) and homolog of Pumilio, Drosophila, 1 (PUM1)) to be the most suited for normalization of RT-qPCR data in both the collected group of human breast tissue samples (ER+ and ER- IBC and normal breast tissue) and the IBCs (ER+/ER-). These genes should be used if employing an averaged normalization strategy. Should finances or limited amount of material only allow a single gene be used for normalization, PUM1 was identified for all of the above mentioned samples as the single best gene.

## Methods

### ▪ Cells

Three ER+ cell lines, T47D (HTB-133, American Type Tissue Culture (ATCC), Manassas, VA), MCF-7 (HTB-22, ATCC) and BrCa-MZ-01 [14] were used. T47D was grown in RPMI1640 with 2 mM L-glutamine, containing 1.5 g/L sodium bicarbonate, 4.5 g/L glucose, 10 mM HEPES, 1.0 mM sodium pyruvate, supplemented with 0.2 U/mL bovine insulin (90%) and 10% fetal bovine serum. MCF-7 were grown in minimum essential medium (Eagle) with 2 mM L-glutamine, containing 1.5 g/L sodium bicarbonate, 0.1 mM non-essential amino acids, 1.0 mM sodium pyruvate, supplemented with 0.01 mg/mL bovine insulin (90%) and 10% fetal bovine serum. BrCa-Mz-01 was grown in DMEM with high glucose, 1% non-essential amino acids, 1% L-glutamine, 1% sodium pyruvate, 1% fetal calf serum and 1% Penicillin/Streptomycin. All were incubated at 37°C, with 95% air and 5% CO_2_. The cells were counted, pelleted by centrifugation at 2.3 × g for 3 min and stored at a concentration of 5 × 10^6 ^cells/mL in MagNa Pure LC mRNA isolation kit I lysisbuffer (Roche, Mannheim, Germany) at -80°C for a maximum of 2 months. The cells were verified as ER+ by immunohistochemistry.

### ▪ Tissue sampling

Eleven ER+ and 4 ER- breast carcinomas from patients undergoing primary surgery at Odense University Hospital were included in the study. Mean age 62 years (range 44–90 years), mean tumor size 26 mm (range 8–45 mm), malignancy grade 1–3. All ER+ tumors were 100% positive for ER as determined by IHC. Three normal breast tissue samples were obtained from breast reductive surgery. A 0.5 × 0.5 cm piece of each sample was snap frozen within 30 minutes of excision in isopentane after covering with TissueTek (Sakura Finetek, Zoeterwoude, The Netherlands) and stored at -80°C until use. Sections of each carcinoma were stained with haematoxylin/eosin to determine the percentage of tumor cells, which was confirmed to be >50% in all instances. Tumor tissue (30 mg) was homogenized in 800 μL lysisbuffer (Roche) using MagNa Lyser Green beads/MagNa Lyser instrument (Roche) for two 10 sec pulses at 6.500 rpm, and stored at -80°C for a maximum of 2 months. The study was approved by the ethical committee of Funen and Vejle County, Denmark.

### ▪ Laser Capture Microdissection (LCM)

To specifically evaluate gene expression in isolated breast cancer cells, one ER+ breast cancer sample underwent LCM to isolate the tumor cells. To avoid amplification of RNA, LCM of 16 tissue slides were pooled, yielding a concentration of 10.72 ng/μL (NanoDrop ND-1000 Spectrophotometer, V3.1 (NanoDrop Technologies, Wilmington, DE)). In brief, cryosections (7 μm) were cut and applied to slides. Pretreatment of the slides was as follows: Sections were thawed for 15–30 sec (without hydration), 70% EtOH was applied for 30 sec, 15–30 sec hydration in DEPC-treated water, haematoxylin-stained by dipping ×3 in solution. Samples were then washed with DEPC-treated water and dipped in autoclaved tap-water to intensify nuclear staining, placed in 70% EtOH for 30 sec, 96% EtOH for 30 sec, 1–2 dip in eosin solution, 96% EtOH for 30 sec, two times 100% EtOH for 30 sec (separate vials) and xylene for 4 min. The slide was then placed in a fume hood for 5 min to air-dry the xylene. LCM was conducted for a maximum of 1 hr to ensure reliability of RNA integrity. LCM was carried out on the PixCell^® ^IIe (Arcturus Bioscience Inc., CA) isolating total RNA using the RiboPure RNA isolation kit (Arcturus) with 50 μL extraction buffer, according to manufacturer's protocol.

### ▪ RNA purification

RNA was purified from 350 μL of lysis buffer containing the cell lines or the homogenized tissue samples by Roche RNA isolation kits for cells or tissue, respectively (MagNa Pure LC RNA isolation kit III tissue and MagNa Pure LC RNA isolation kit – high performance) using the MagNa Pure Robot (Roche). This system uses magnetic beads to isolate total RNA. Concentration and purity (260/280 ratio) were measured in duplicate by the NanoDrop (NanoDrop Technologies), achieving a mean concentration of 16.9 ng/μL (3.22 – 60.45 ng/μL) and a 260/280-ratio of 1.99 ± 0.2 (SD). RNA obtained from cell cultures and frozen tissue was not significantly degraded, as determined by gel electrophoresis (data not shown).

### ▪ cDNA synthesis

Total RNA (10 μL) was reverse-transcribed to cDNA using random 9 oligonucleotide primers at 25 μM per reaction. RNA and primers were incubated for 5 min at 70°C and subsequently placed on ice. A reaction mix consisting of 1 mM dNTPs, 1 Unit/μL RNase Inhibitor (Roche), 10 Unit/μL Reverse Transcriptase (Invitrogen Life Technologies, Paisley, UK) and First Strand Buffer ×5 (Invitrogen) was added and incubated for 10 min at 25°C, followed by 45 min at 37°C, and 5 min at 95°C.

### ▪ Quantitative real-time RT-PCR (RT-qPCR)

The TaqMan^® ^Gene Expression Assays (Applied Biosystems, Foster City, CA, USA) were used for all reactions for the genes shown in table 1. The existence of pseudogenes was investigated by BLAT search [15], analyzing the sequence of the entire potential amplicon, as the precise cDNA sequence was unavailable. This BLAT search was devised as the assay location ± amplicon size provided + around 10 bp at each end. The presence of pseudogenes, and thereby possible cross-reactivity with gDNA, was defined as regions of >95% identity for an approximately equal length to the potential amplicon. The 25 μL RT-qPCR reaction mix contained 1× TaqMan universal mastermix, 1× assay mix including primers and probe, 5 μL cDNA, gDNA or dilution series, and RNase/DNase-free water. The reactions were run for 2 min at 50°C, 10 min at 95°C, followed by 50 cycles of 15 seconds at 95°C and 1 min at 60°C. All RT-qPCR reactions were performed on the ABI 7900 HT system (Applied Biosystems) and were measured in replicate (triplicate or duplicate) to ensure methodological reproducibility, which was within a maximum of 0.24 Ct's. The detection limit was set at Ct 41.

**Table 1:** Summary of candidate reference genes.

**Symbol**	**Gene name (Assay ID)**	**Function**	**Location**	**Assay location (amplicon size)**	**PG**^¥^	**A/CB **^#^
RPLP0 NM_053275.3	Ribosomal protein, large, P0 (Hs99999902_m1)	Structural protein of ribosomes	12q24.2	325* ^• ^(105)	+	(-/+)
TBP NM_003194.3	TATA box binding protein (Hs00427620_m1)	Transcription factor; DNA-dependent transcription initiation from Pol II promoter	6q27	730 (91)	-	(-/-)
PUM1 NM_001020658.1	Pumilio homolog (Drosophila) (Hs00982765_m1)	RNA binding; translation factor.	1p35.2	1757 (68)	-	(-/-)
ACTB NM_001101.2	β-actin (Hs99999903_m1)	Cytoskeletal structural protein involved in various types of cell motility	7p22-p12	36* ^× • ^(171)	+	(-/+)
GUS-B NM_000181.1	Glucoronidase, beta (Hs99999908_m1)	Hydrolase; carbohydrate metabolism.	7q21.11	1816 (81)	-	(-/NI)
ABL1 NM_005157.3	v-abl Abelson murine leukemia viral oncogene homolog 1 (Hs00245443_m1)	Protein kinase; regulation of cell cycle, mismatch repair, DNA damage response	9q34.1	85 (54)	-	(-/NI)
GAPDH NM_002046.3	Glyceraldehyde-3-phosphate dehydrogenase (Hs99999905_m1)	Oxidoreduct-ase; glucose metabolism	12p13.31-p13.1	158* ^× • ^(122)	-	(+/NI)
B2M NM_004048.2	β-2-microglobulin (Hs00187842_m1)	Major histocompati-bility complex antigen class 1 receptor activity	15q21-q22	128 ^• ^(64)	-	(-/NI)

NI: not investigated.

^¥^: PG = pseudogenes. BLAT search to identify pseudogenes of potential amplicons. See materials and methods for criteria.

^#^: Experimentally observed to A = amplify gDNA or CB = competitive binding of primers or probe with gDNA.

*: amplicon does not cross exon-exon boundaries.

×: known pseudogenes exist. These genes have been designed to amplify a region of the 5'UTR.

^•^: SNP(s) are located under a primer or probe sequence

Since we used assays-on-demand, we wanted to verify performance of the primer and probe sets. The reference genes were methodologically analyzed by a 10-fold dilution series of cDNA from MCF-7 in water (1-1:100.000) to ensure optimal amplification efficiency. In addition, the 4-group reference genes for ER+ IBC were analyzed by a dilution series in MCF-7 derived gDNA (1-1:10.000) to test for competitive binding. TissueTek and the tissue itself were also tested for inhibitory components. To examine the effect of TissueTek on the PCR, a 2-fold dilution series was conducted with cDNA from MCF-7 and TissueTek (1:2 – 1:16) treated identically to patient tissue from freezing in isopentane to cDNA synthesis (included), using the expression assay for β2M. Only a minimal increase, i.e. inhibition of the PCR reaction, was observed for the 1:2 dilution, raising the Ct by 0.7. It was therefore concluded that TissueTek does not need to be macro-dissected from tissue sections since it is never found in such excess in patient tissues. A 10-fold dilution series was conducted on one patient's cDNA using the reference gene TBP to test for inhibitory components in the tissue. No inhibition was observed (amplification efficiency 2.0). Calculations for the amplification efficiency were = $\sqrt[{\alpha }]{10}$, where α is the slope of the standard curve obtained by a dilution series, plotted as Ct-value (y-axis, linear values) versus the dilution (x-axis, logarithmic values).

### ▪ PCR

A PCR was performed with the same program as RT-qPCR for the 4-group reference genes for ER+ IBC with cDNA from one patient (no. 9) and the MCF-7 cell line. The products were run on a 3% NuSieve GTG agarose gel (Cambrex Bioscience Rockland Inc., Rockland, ME) with 20 μg EtBr (Sigma, St. Louis, MO) in 1× TBE buffer (Sigma) at 5 V/5 min followed by 100 V/45 min (BioRad, Hercules, CA) along with a PCR marker (New England BioLabs Inc., Ipswich, MA).

### ▪ Immunohistochemistry (IHC)

The NCL-ER-6F11 antibody (Novocastra Ltd., Newcastle, UK) was used for ER detection. Antigen retrieval was performed using Tris-EGTA, pH 9, incubated overnight at 60°C, followed by blockage of endogenous peroxidase by H_2_O_2 _for 10 min. The primary antibody (1:100) was incubated for 60 min and detected by PowerVision (Immunovision Technologies, Fullerton, CA, USA), followed by nuclear counter-staining with Mayers Haematoxylin for 2 min. All immunostainings were performed using the Autostainer (Dako) and known positive and negative controls were included. The steroid receptors were scored positive if > or = 10% of the tumor cells had nuclear staining.

### ▪ Data analysis and statistics

The RT-qPCR raw data was analyzed using the SDS software, vers. 2.1 (Applied Biosystems). Descriptive statistics were conducted using box-plots (STATA, vers. 9, TX). The RT-qPCR raw data was converted to linear values compatible with the geNorm and NormFinder programs by qBase, v.1.3.4. (Visual Basic application tool for Microsoft Excel available on the Internet) [16]. For stability comparisons of candidate reference genes, the software geNorm, vers. 3.4 [12] (Visual Basic application tool for Microsoft Excel, available on the Internet) and NormFinder [13] (a Microsoft Excel Add-in available on the Internet) were used according to author's recommendations. In addition, the option in NormFinder to define sub-groups was applied for ER+ IBC patients for important prognostic factors such as number of positive lymph nodes, tumor size and malignancy grade. The sub-groups were defined as follows: Lymph nodes – 3 groups; 0 (n = 3), 1–3 (n = 5) and >4 (n = 3) lymph nodes with metastasis. Tumor size – 2 groups; ≤ 20 mm (n = 5) and > 20 mm (n = 6). Malignancy grade – 3 groups; grade 1 (n = 3), grade 2 (n = 4) and grade 3 (n = 4).

## Results

### Selection of candidate genes

Eight genes were selected for investigation to identify the most stably-expressed reference gene to be used in RT-qPCR studies of breast cancer, with specific emphasis on ER+ IBC. The 8 candidates were selected based on previous reports on RT-qPCR studies examining IBCs (RPLP0, TBP and PUM1) [17-19] or were commonly used reference genes (ACTB, GUS-B, ABL1, GAPDH and B2M) (Table 1).

### Performance of the RT-qPCR assays

The 8 reference genes were tested for possible amplification of genomic DNA to ensure that the observed amplification was not the result of genomic DNA in the RNA-purified sample. No amplification was observed for the genes, with the exception of GAPDH, which weakly amplified genomic DNA from MCF-7 cells at an absolute Ct' of 37.0. The PCR assays were assessed since they contained pre-designed primer-probe sets (Table 2) and the primer and probe sets were confirmed to have performed perfectly, with an amplification efficiency of 1.9–2.0. In addition, the specificity was investigated by PCR and gel separation, confirming an expected single band at the reported amplicon size, as shown in Table 1.

**Table 2:** Performance of the qPCR. Standard curve results are summarized and the amplification efficiency calculated.

	Dilutant					
	
	**Water**			**gDNA**		
	
**Genes**	Slope	R^2^	Amplification efficiency	Slope	R^2^	Amplification efficiency
PUM1	-3.49	0.998	1.9	-3.47	0.998	1.9
TBP	-3.36	0.988	2.0	-3.27	0.998	2.0
RPLP0	-3.56	0.999	1.9	-5.70	0.924	1.5*
ACTB	-3.52	0.999	1.9	-4.91	0.940	1.6*
GAPDH	-3.27	0.999	2.0			
B2M	-3.45	0.998	2.0			
ABL	-3.41	0.999	2.0			
*GUS-B*	-3.33	0.998	2.0			

Dilution in gDNA was conducted to investigate competitive binding for the 4-group reference genes for ER+ IBC.

*: theoretical amplification efficiencies. The actual efficiency cannot be determined since the standard curves were flawed, as observed by the low coefficient of correlation (R^2^)

### Descriptive statistics

**Figure 1:**
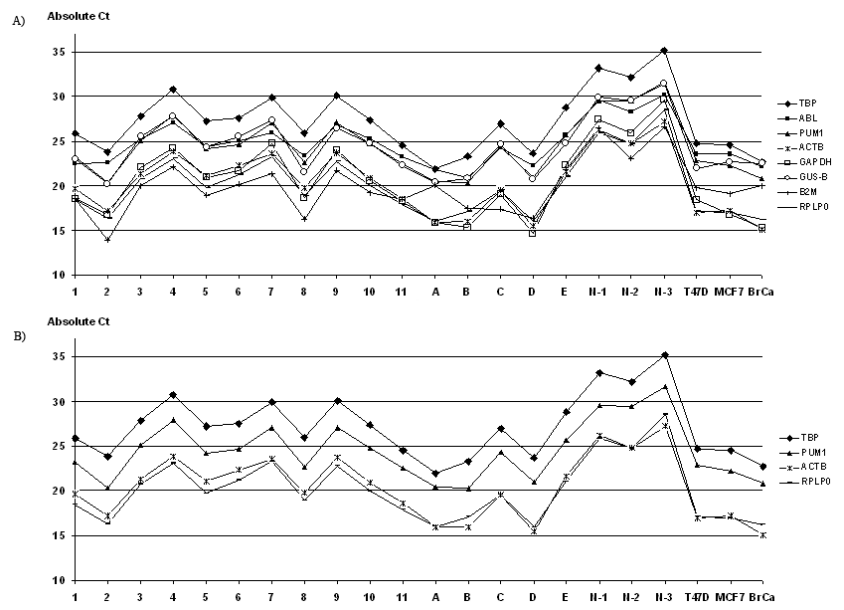
**Absolute Ct values of 11 ER+ (1–11) and 4 ER- (A-E) IBCs, 3 normal breast tissue samples (N-1 – N-3) and 3 ER+ cell lines (T47D, MCF-7 and BrCa)**. All genes were investigated using the same RNA/cDNA batch per sample. A) all investigated reference genes. B) the 4-group reference genes for ER+ IBC.

**Figure 2:**
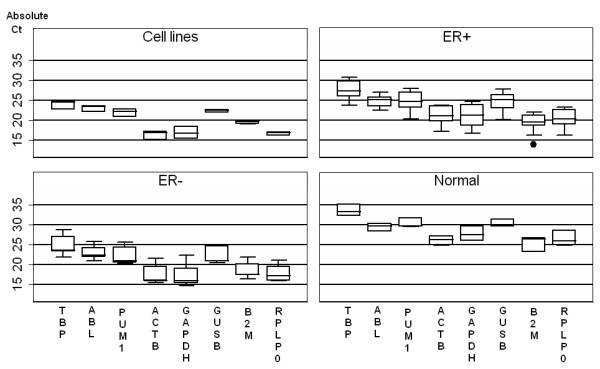
**Box plot of the absolute Ct values of the 8 reference genes investigated on the 4 sub-groups**. The same batch of RNA/cDNA was used for each patient across the genes.

The absolute expression levels of the 8 reference genes in the 11 ER+ IBCs (Fig. 1) were observed spanning on average a Ct value of 7.2 between the most abundant (β2M) and least abundant genes (TBP). Interestingly, TBP was consistently the least abundant gene in all sample types investigated, whereas the most abundant gene varied between sample groups. For ER+ tumors and normal breast tissue, β2M was the most abundant, for ER- tumors and cell lines it was ACTB and GAPDH, (Fig. 2).

### Identification of optimal reference genes

Initially, the absolute Ct values for the samples were graphed to visualize the stability of the genes (Fig. 1). However, this graphic representation clearly illustrated that an objective assessment of the genes without the aid of statistical analysis was not possible. The two most commonly utilized computer programs developed to identify the most suitable reference genes in various applications are geNorm [12] and NormFinder [13]. These programs were used in our study to analyze expression of the candidate reference genes in different sample types. GeNorm identifies the optimal genes and how many are required for optimal stability by pair-wise comparisons, whereas NormFinder enables identification of the single best genes as a ranking order. Table 3 summarizes the results of this analysis and shows the optimal reference gene(s) of the individual sample groups and combinations thereof. When examining the 18 patient tissue specimens (ER+/ER- IBC/normal) or the breast cancer tissue specimens alone (ER+/ER- IBC), the 3-gene combination of RPLP0, TBP and PUM1 were consistently identified by geNorm to exhibit the highest degree of stability. For ER+ IBC samples alone, geNorm identified the same set of 3 genes mentioned above, but a fourth gene (β-actin) was recommended for increased stability. When the cell lines were also included, a fifth gene (GUS-B) was additionally recommended to obtain the highest degree of stability. NormFinder uniformly identified the PUM1 gene as the single most stable gene for all sample combinations, as well as the ER+ IBC group alone (Table 3).

**Table 3:** Summary of top candidate reference genes identified by geNorm and NormFinder. For the geNorm data, the optimal number and identity of reference genes proven necessary by a Pairwise variation below 0.15, as recommended by Vandesompele et al. [12], is provided in the order given by the program. The stated genes should be viewed per program, and the combination of genes provided by geNorm cannot be used as single entities as optimal reference genes. The number of patients included was: ER+ (n = 11), ER- (n = 4), normal (n = 3) and ER+ cell lines (n = 3)

	**geNorm**	**NormFinder**
**ER+, ER-, normal and cell lines**	RPLP0/TBP, PUM1, GUS-B and β-actin	PUM1 PUM1/RPLP0
**ER+, ER- and normal tissue**	TBP/PUM1 and RPLP0	PUM1 PUM1/RPLP0
**ER+ and ER-**	TBP/PUM1 and RPLP0	PUM1 PUM1/RPLP0
**ER+**	RPLP0/TBP, PUM1 and β-actin	PUM1
**ER-**	PUM1/GUS-B and RPLP0	β-actin
Normal*	PUM1/GUS-B and TBP	TBP
**ER+ cell lines**	TBP/PUM1	ABL

* initially geNorm identified GAPDH/RPLP0 and TBP as the best candidates. GAPDH was found earlier to cross-react to pure genomic DNA and was therefore removed from the panel.

The 4-group reference genes identified for ER+ IBC, RPLP0, PUM1, TBP and β-actin (geNorm), were additionally tested for competitive binding to genomic DNA by a dilution series in genomic DNA to examine the necessity of DNase treatment of purified RNA. Reduced amplification efficiencies for RPLP0 and ACTB were observed (Table 2), underscoring the importance of DNase treatment in these two assays.

### Evaluation of possible influence of prognostic factors on reference gene expression in ER+ IBCs

**Figure 3:**
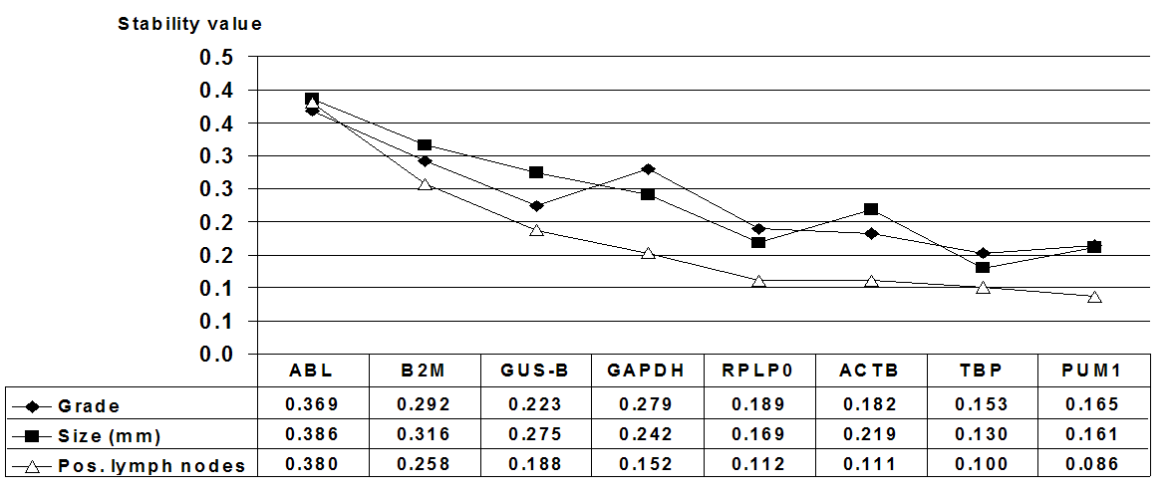
**Stability value (NormFinder) for ER+ IBCs with sub-grouping based on prognostic factors**. The sub-groups were defined as follows: Grades 1, 2, and 3. Size: above/below 20 mm. Positive lymph nodes: 0, 1–3 and >4. GAPDH should be used with caution since the primer-probe set used in this study have shown cross-reaction with pure genomic DNA.

NormFinder has the option of defining groups within the samples, and was utilized to confirm that important prognostic parameters such as the number of positive lymph nodes, tumor size and malignancy grade did not influence selection of the identified optimal reference genes for the ER+ IBC group (Fig. 3). As relatively few samples were analyzed in this study, this evaluation of subgroups should be viewed as representing an interesting tendency needing further investigation.

### Laser capture microdissection of ER+ tumor cells

**Figure 4:**
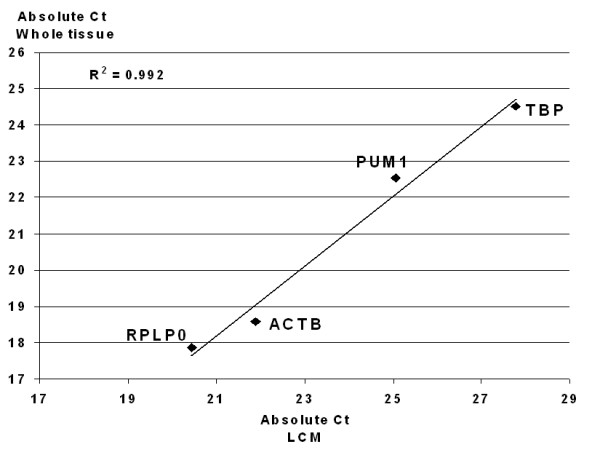
**Comparison of laser capture microdissected tumor cells and whole-tissue sections**. The correlation of the gene expression of the 4-group reference genes for laser capture microdissected (LCM) tumor cells vs. whole tissue sections of ER+ IBC was investigatedby Pearson analysis.

To specifically examine the reference gene expression of ER+ IBC cells from patient tissue, tumor cells from one ER+ IBC patient were laser capture microdissected. The analysis showed nearly parallel expression to whole-tissue from the same tumor using the 4-group reference genes for ER+ IBC of TBP, RPLP0, PUM1 and ACTB (Fig. 4), as observed by a Pearson correlation of 0.992. β-actin and TBP had an absolute Ct difference of 3.2, whereas PUM1 and RPLP0 had an absolute difference of 2.5, giving an absolute variation of only 0.7 Ct's.

## Discussion

The importance of reference genes specific to a particular experimental set-up for normalization of RT-qPCR data is well-recognized. However, the use of multiple reference genes rather than a single gene remains a matter of debate, although it is widely accepted that multiple genes minimize the influence of minor fluctuations [12].

We report herein the first systematic evaluation of several conventional and novel potential reference genes for accurate normalization of real-time RT-qPCR data in breast cancer. The use of a liquid handling robot, high quality real-time PCR reagents and commercially available expression assays designed to maximize PCR efficiency and specificity allows trustworthy comparison of genes. The reference genes were evaluated using ER+ and ER- IBC, normal breast tissue and ER+ cell lines, and the data were analyzed using 1) descriptive statistics (i.e. boxplot and absolute value comparisons), 2) geNorm and 3) NormFinder programs that identified the most stably expressed genes. The top 4 genes were also evaluated using laser capture microdissected material of an ER+ tumor to compare expression in whole-tissue vs. isolated cancer cell expression.

The combination of the three genes TBP, RPLP0 and PUM1 were repeatedly identified as the optimal reference genes with the least variation among patient samples (for IBC, both with or without normal tissue) by GeNorm. A fourth, β-actin, was additionally required for the ER+ IBC samples. Evaluating the samples as single entities, (i.e. ER+ IBC, ER- IBC, normal or cell lines) rather than combinations thereof did not significantly alter the most optimal reference genes identified.

Various methods to compare RT-qPCR data have been used since the initial development of real-time PCR in 1996 (reviewed by Hugget et al. in 2005 [5]). While it is preferable to use internal reference genes, it is important to note that some traditional reference genes may be regulated in certain settings, e.g. B2M in brain cortices of human chronic alcoholics [6,20] and GAPDH in MCF-7 cells treated with estradiol [7]), which underscores the importance of identifying appropriate reference genes prior to the start of a study. The availability of easy-to-use, dependable and freely available computer programs has made it easier to identify optimal normalizers. The first of these computer programs, geNorm [12], was reported in 2002, shortly followed by NormFinder [13] in 2004. Other statistical models to identify optimal reference genes have now become available [18,21,22], but since these are not freely available or easy to use, we focused on geNorm [12] and NormFinder [13] in this study. A third freely available program, BestKeeper, uses Pearson correlation to calculate stable genes based on raw data [22]. This approach may be useful to narrow down a search if no specific genes are known to be plausible candidates. More advanced statistics are needed to rank the genes if several are identified as good candidates, as provided by geNorm and NormFinder.

Despite the use of programs to identify optimal normalizers, the difficulty of distinguishing between genes identified as stable and those being co-regulated in a given experimental set-up remains. In addition, since the entire biological function of most genes is not known, differentiation of these groups cannot be delineated theoretically. To decrease the likelihood of identifying a group of optimal reference genes that are co-regulated, reference genes located on different chromosomes and involved in different basic cellular processes should be included. It has been argued that NormFinder, with its model-based approach that permits definition of sub-groups within the investigated samples, is more precise than the pairwise comparison approach used by geNorm [12], since the former identifies co-regulated genes based on the similarity of their expression profiles, and genes showing no difference between the sub-groups would be excluded early in the pairwise comparison. This sub-group definition option in NormFinder becomes most noteworthy when looking for optimal reference genes across samples that can differ in their hormonal states (ER+/ER- IBC) or pathogenesis (cancer/normal tissue). Accordingly, we estimate that in our study, where we sought to identify the optimal reference genes within a homogenous population of primarily ER+ IBC, geNorm and NormFinder without using the option of sub-grouping, were equivalent in performance, and therefore comparable. This assumption was confirmed by the fact that both programs identified the same top 4 genes, TBP, RPLP0, PUM1 and β-actin, although in differing rank order, for all patient samples, both in combination (geNorm) and as single entities (NormFinder), strongly suggesting that these genes are not influenced by hormonal status or general pathogenesis. Furthermore, defining prognostic sub-groups (number of positive lymph nodes, malignancy grade or tumor size) for the ER+ patients in NormFinder identified the same top 4 genes with the smallest variation. Although the low sample size does not allow a definitive conclusion, it does show a tendency meriting further investigation. An increase in aggressiveness has been shown to influence which optimal gene to normalize against for breast cancer cell lines [23], however this did not seem to be the case for the patient samples and genes evaluated in this study.

Another very important consideration when choosing reference genes is that the design of the primers and probes accounts for possible pseudogenes or single-nucleotide polymorphisms (SNPs). In addition, to prevent amplification of contaminating gDNA, the amplicon should cross exon-exon boundaries. An amplicon including an intron is quite long and usually results in an incomplete transcript that does not contain the sequence for the reverse primer, thereby preventing further amplification.

It is possible to use a gene that has pseudogenes if special care is taken when selecting the cDNA region to be amplified, ensuring that the primers and probe sequences do not have high homology to any gDNA sequences. This can be investigated by a BLAT search [15], which was conducted on the genes examined in this study (table 2), and showed that the percentage of homology and the span of the homologous region was acceptable. As this is a theoretical search, the primers and probe set must be tested for reactivity to gDNA. Of the 4 selected genes, ACTB and RPLP0 have known pseudogenes, but did not amplify pure gDNA, which was circumvented by placing the primers and probe to target the 5'UTR. However, competitive binding could not be avoided, as seen by the decreased amplification efficiency when conducting a standard curve using serial dilutions of cDNA in gDNA (Table 2). Such decreased amplification efficiency is observed when competitive binding occurs as the primer or probe cross-reacts with gDNA when in excess, and thus the primers or probe become limiting factors in the reaction. The oligonucleotide binds to the wrong sequence and is subsequently elongated, rendering the primer or probe useless and unavailable for amplification of the desired cDNA sequence. In addition to choosing the optimal reference gene(s) to normalize with, it is important to ensure that only logarithmic amplification of the target occurs in the reaction. If DNase treatment is omitted, and the primers or probe bind to gDNA, linear amplification will occur, resulting in a flawed Ct value if gDNA is in excess.

This pre-PCR step of DNase treatment is crucial when employing reference genes known to cross-react with gDNA in a competitive manner, since comparable amplification efficiencies are required to compare reference and target genes. If this is not the case, a shift in the amplification curve may be introduced that influences the ΔCt values. This can be exemplified by a primer-probe set with a reduced efficiency of 1.5 compared to the ideal of 2.0, resulting in a 10^4 ^difference in product amount after 30 cycles. If the primer and probe set amplifies gDNA with resulting emission of fluorescence, they should not be used as an assay for detection of either reference genes or target genes even if the sample is treated with DNase. DNase treatment is not 100%, and presence of gDNA, although minor, will result in an incorrect, slightly higher Ct value due to logarithmic amplification. Competitive binding to gDNA, on the other hand, does not exclude the use of the primer and probe set, as the DNase treatment will decrease the amount of gDNA to such a degree that the cDNA will be in significant excess and the use of primers or probe for unspecific sequences will be very small. In addition, there will be no emission of fluorescence to affect the Ct value. Competitive binding will therefore not be an issue if DNase treatment is included. Thus, the importance of including a DNase treatment step when purifying RNA for RT-qPCR, including the 4 identified reference genes in this study, is apparent. In addition, special care must be taken when using the ACTB or RPLP0 genes as reference genes to be sure no gDNA amplification occurs, since known pseuodogenes exist. This would lead to over-estimation of the amount of input mRNA and imprecise normalization of the target genes. For ACTB and RPLP0, an amplicon crossing an exon-exon boundary would not implicitly decrease the likelihood of amplification of gDNA due to pseudogenes. When a DNase treatment step was included in this study, the competitive binding for these 2 of the top 4 reference genes was not an issue, and as no amplification was seen for pure gDNA, the Ct values measured for the top 4 reference genes were specifically due to mRNA expression.

The presence of SNPs in most of the reference genes investigated should not be an excluding factor and SNPs probably exist in all genes. The presence of SNPs in candidate reference genes should be revealed during testing of the stability of the genes using the computer programs, wherein the gene would appear up- or down-regulated compared to other, stably-expressed, genes due to the varying amplification efficiencies caused by mismatched base pairs between the SNP-site(s) and the primers or probe. The varying Ct values should result in elimination of the gene as a suitable candidate.

The identification of reference genes is most often conducted in a limited number of samples and the genes should be re-evaluated after the end of the experiments, preferably by one of the objective computer programs including both the target and reference gene(s). It could also be done by viewing the ΔCt between the reference genes across all samples (as illustrated in Fig. 1), a method that should result in a very small standard deviation.

Finally, the expression of the selected genes was evaluated in consort with the various tissue components. Tissue sections of solid tumors contain various cell populations, such as stroma, lymphocytes and tumor cells. Only minor variations in gene expression of LCM-isolated tumor cells compared to whole tissue was observed when examining the 4-group reference genes for ER+ IBC. The Pearson correlation was 0.992 (Fig. 4). The absolute difference between tumor cells and whole tissue was 0.7 Ct, nearly within the accepted technical variation of 0.5 Ct for RT-qPCR.

## Conclusion

The consistent identification of TBP, RPLP0 and PUM1 provides strong support for their use in RT-qPCR data normalization of normal and malignant human breast samples. Based on geNorm, a fourth reference gene was required when examining ER+ IBCs alone (β-actin). In addition, the PCR characteristics were thoroughly evaluated, emphasizing the need for a DNase pre-PCR treatment step.

## Abbreviations

ABL: beta v-abl Abelson murine leukemia viral oncogene homolog 1; ACTB: β-actin; AI: Aromatase inhibitor; B2M: β-2-microglobulin (B2M); ER: Estrogen receptor; GAPDH: Glydecaldehyde-3-phosphate-dehydrogenase; gDNA: Genomic DNA; GUS-B: Glucoronidase; IBC: Invasive breast cancer; IHC: Immunohistochemistry; KRT: Cytokeratin gene; PUM1: Homolog of Pumilio, Drosophila, 1; RT-qPCR: Quantitative real-time reverse transcriptase polymerase chain reaction; RPLP0: Ribosomal protein, large, P0; TBP: TATA-box binding protein.

## Competing interests

The author(s) declare that they have no competing interests.

## Authors' contributions

Conception, design and data acquisition of data was done by MBL, under the supervision of NP and HJD. All handling of patient samples and IHC analysis was conducted in collaboration with AVL. MBL drafted the manuscript, which was revised by HJD. All authors have given their final approval of the version to be published.

## Pre-publication history

The pre-publication history for this paper can be accessed here:


